# From Pseudo-Surgical Abdomen to Renal Amyloidosis: Delayed Diagnosis of Familial Mediterranean Fever in an Adolescent

**DOI:** 10.7759/cureus.111557

**Published:** 2026-06-26

**Authors:** Hind Azal, Sophia Yahyaoui, Said Kaddouri, Mohamed Zyani, Hassan Qacif

**Affiliations:** 1 Internal Medicine, Centre Hospitalier Universitaire Mohammed VI, Marrakech, MAR; 2 Internal Medicine, Avicenne Military Hospital/Cadi Ayyad University, Marrakech, MAR; 3 Internal Medicine, Ibn Sina Military Hospital, Marrakech, MAR

**Keywords:** aa amyloidosis, colchicine-resistant, familial mediterranean fever, interleukin-1 inhibitors, mefv m694v mutation

## Abstract

Familial Mediterranean fever (FMF) is an autosomal recessive autoinflammatory disease characterized by recurrent febrile episodes and serositis. Delayed diagnosis remains a major concern, as it may lead to severe complications, particularly AA amyloidosis.

We report the case of a male patient, born to a consanguineous family, with recurrent episodes of fever and abdominal pain since early childhood, initially leading to an unnecessary appendectomy. The diagnosis of FMF was established at the age of 13 following the onset of nephrotic syndrome. Renal biopsy confirmed AA amyloidosis, and genetic testing revealed a homozygous M694V mutation in the *MEFV* gene. Despite colchicine therapy, persistent disease activity required initiation of anakinra, later switched to canakinumab due to ongoing subclinical inflammation, evidenced by an elevated serum amyloid A level with normal C-reactive protein.

This case highlights the consequences of delayed diagnosis in FMF, the importance of early recognition in patients with recurrent abdominal pain, and the role of serum amyloid A in disease monitoring. It also illustrates intrafamilial phenotypic variability and the need for therapeutic escalation in colchicine-resistant disease.

## Introduction

Familial Mediterranean fever (FMF) is the most common monogenic autoinflammatory disease, predominantly affecting populations from the Mediterranean basin. It is caused by mutations in the *MEFV* gene and is characterized by recurrent, self-limited episodes of fever and serositis (peritonitis, pleuritis), or arthritis [1]. 

Despite its typical clinical features, FMF remains underdiagnosed, particularly in children, due to nonspecific presentations. Abdominal attacks may mimic acute surgical conditions, often leading to unnecessary procedures such as appendectomy. Delayed diagnosis results in prolonged uncontrolled inflammation and significantly increases the risk of developing AA amyloidosis, the most severe complication of the disease. AA amyloidosis is caused by the extracellular deposition of serum amyloid A (SAA) protein, an acute-phase reactant that accumulates as a result of chronic, persistent inflammation. This distinguishes it from AL amyloidosis, which is driven by misfolded immunoglobulin light chains in the context of plasma cell dyscrasias, and from hereditary amyloidosis subtypes caused by mutations in specific proteins such as transthyretin. In FMF, uncontrolled interleukin-1 (IL-1)-mediated inflammation leads to sustained SAA elevation, which over time promotes amyloid fibril formation and preferential deposition in the kidneys [1].

Several factors have been associated with an increased risk of progression to AA amyloidosis in FMF, including male sex, early disease onset, homozygous M694V genotype, geographic origin, and, most importantly, inadequate inflammatory control reflected by persistently elevated SAA levels [1].

We report a case of colchicine-resistant FMF, revealed by AA amyloidosis, in a patient with a homozygous M694V mutation, highlighting the consequences of delayed diagnosis, the importance of SAA monitoring for detecting subclinical inflammation, intrafamilial phenotypic variability, and the role of IL-1 inhibition in managing refractory disease.

## Case presentation

A three-year-old male, born to a first-degree consanguineous union, initially presented with recurrent episodes of fever associated with severe abdominal pain, occurring every few weeks, lasting approximately five days, and resolving completely and spontaneously without residual symptoms between episodes. The attacks were not associated with chest pain, joint involvement, or skin lesions. At the age of five, an acute episode of severe generalized abdominal pain, fever, and elevated C-reactive protein (CRP) suggestive of peritonitis led to an appendectomy; however, no definitive surgical cause was identified. Similar episodes recurred postoperatively, supporting a non-surgical etiology, and these manifestations remained undiagnosed for several years, managed symptomatically with analgesics and antispasmodics. Subsequent evaluations, including digestive endoscopy and enteroscanner when systemic involvement was suspected, did not reveal any significant abnormalities.

At the age of 13, the patient presented with generalized edema. Upon examination, blood pressure was at 100/60 mmHg. Urinalysis revealed significant proteinuria without hematuria or leukocyturia, and no evidence of urinary tract infection. Laboratory investigations confirmed nephrotic-range proteinuria quantified at 11 g per 24 hours, with hypoalbuminemia of 22 g/L and total serum protein of 52 g/L, while renal function remained preserved. Biological evaluation demonstrated a marked inflammatory syndrome, with elevated CRP (124 mg/L) and an accelerated erythrocyte sedimentation rate (100 mm/h). Serum protein electrophoresis was consistent with nephrotic syndrome. Complement levels (C3, C4, and CH50) were decreased. The finding of hypocomplementemia was atypical in the context of FMF-associated AA amyloidosis. Extensive investigations were performed to exclude alternative causes of complement consumption, including systemic lupus erythematosus, infection-related glomerulonephritis, cryoglobulinemia, and anti-neutrophil cytoplasmic autoantibody (ANCA)-associated vasculitis. Antinuclear antibodies, anti-extractable nuclear antigen antibodies, and antineutrophil cytoplasmic antibodies were negative, and infectious investigations were unrevealing. Renal biopsy did not demonstrate features suggestive of an immune-complex-mediated glomerulonephritis. Although the mechanism underlying the transient complement consumption remains uncertain, which may reflect undercurrent immune activation, no supporting disease was found. However, the overall clinical, genetic, and histopathological findings strongly supported AA amyloidosis secondary to FMF. Given the presentation of nephrotic syndrome associated with an inflammatory profile and hypocomplementemia, an underlying glomerular disease was suspected, and the patient underwent a renal biopsy followed by empirical pulse therapy with methylprednisolone (500 mg/day for three days) while awaiting histological diagnosis. However, no significant clinical or biological improvement was observed. Renal biopsy confirmed the presence of amyloid deposits, demonstrated by Congo red staining with characteristic apple-green birefringence under polarized light. The deposits involved glomeruli, vascular walls, and the interstitium, consistent with renal amyloidosis.

Amyloid subtyping was performed on renal tissue using immunofluorescence, demonstrating AA-type amyloid deposits through positive staining with anti-SAA antibodies. This was consistent with the clinical context of long-standing systemic inflammation due to FMF. Based on this histopathological typing, further investigations for monoclonal gammopathy, including serum and urine immunofixation, serum free light chains, and bone marrow evaluation, were not performed.

Representative histological images were not available for inclusion in this report, as the biopsy was performed at an external institution and archival material could not be retrieved. In the absence of an autoimmune etiology and given the presence of AA amyloidosis, a hereditary autoinflammatory disease was suspected. The family history was notable for the parental consanguinity and a sister who had died at the age of 16 from end-stage renal disease of unknown origin. Genetic testing identified a homozygous M694V mutation in the *MEFV* gene, confirming the diagnosis of FMF. The diagnosis was further supported by the Tel-Hashomer criteria, with the patient fulfilling major criteria including recurrent febrile episodes with peritonitis, as well as multiple supportive criteria including early age of onset, parental consanguinity, and spontaneous resolution of attacks. Colchicine therapy was initiated following diagnosis. Subsequently, a familial genetic investigation was performed, revealing both parents to be heterozygous carriers of the mutation. Among the siblings, one sister was found to carry the same homozygous M694V mutation and exhibited a milder, previously unrecognized clinical phenotype that achieved remission with colchicine alone, while another sibling was heterozygous and remained asymptomatic. Our patient was therefore identified as the index case leading to the diagnosis within the family. In retrospect, the history of a deceased sister who developed end-stage renal disease at the age of 16 raises a strong suspicion of undiagnosed severe FMF complicated by possibly AA amyloidosis. After nearly six months, the patient continued to experience recurrent febrile episodes and abdominal pain, consistent with colchicine-resistant disease. Treatment was therefore escalated to anakinra at a dose of 3 mg/kg/day, resulting in partial clinical improvement. The patient was subsequently lost to regular follow-up, with irregular adherence to medical monitoring despite ongoing treatment.

At the age of 16, he was readmitted to the Department of Internal Medicine for recurrence of abdominal pain. On admission, he was afebrile and hemodynamically stable. Physical examination was unremarkable, with a soft, non-tender abdomen. Serial SAA measurements documented persistently elevated levels throughout the treatment period. Before initiation of anakinra, SAA was 26 mg/L (reference range <10 mg/L). At age 15, despite full adherence to anakinra at the recommended dose of 3 mg/kg/day, SAA decreased only partially to 18 mg/L, reflecting an incomplete therapeutic response. At age 16, SAA had risen again to 22 mg/L despite normal CRP levels, confirming persistent subclinical inflammatory activity. Serum lipase was within normal limits. Abdominal ultrasound and computed tomography confirmed a mild-to-moderate peritoneal effusion and excluded pancreatitis. Liver function tests were within normal limits, arguing against a hepatic etiology. Infectious investigations, including tuberculosis screening, were unrevealing. No features suggestive of malignancy were identified on imaging. Ascitic fluid analysis was attempted; however, the volume of fluid was insufficient for sampling. Given the absence of alternative explanations and the overall clinical context of a known autoinflammatory disease with established AA amyloidosis, the peritoneal effusion was attributed to ongoing FMF-related serosal inflammation. Renal function remained preserved, with an estimated glomerular filtration rate of approximately 86 mL/min and normal serum creatinine levels. However, persistent nephrotic-range proteinuria was noted, estimated at 4 g per 24 hours. Evaluation for systemic amyloid involvement, including echocardiography, enteroscanner, and colonoscopy, did not reveal any cardiac or gastrointestinal abnormalities. The elevated SAA level, together with the presence of peritoneal effusion suggestive of ongoing serosal inflammation, indicated persistent subclinical disease activity despite treatment with anakinra. Given the established role of sustained SAA elevation in the progression of AA amyloidosis and the patient's history of biopsy-proven renal amyloidosis, treatment was escalated to canakinumab in an effort to achieve more complete suppression of inflammation and reduce the risk of further amyloid deposition. At the time of manuscript revision, canakinumab 150 mg subcutaneously every four weeks had been initiated only recently. Follow-up is currently ongoing, and sufficient post-treatment data are not yet available to assess its effects on inflammatory markers, proteinuria, renal function, or long-term disease control. Table 1 summarizes the longitudinal clinical, biological, and therapeutic course of the patient. 

**Table 1:** Longitudinal clinical, biological, and therapeutic course --: data not available; SAA: serum amyloid A; CRP: C-reactive protein; eGFR: estimated glomerular filtration rate; SC: subcutaneous. Reference range: SAA <10 mg/L, CRP <5 mg/L, eGFR >90 mL/min, albumin 35-50 g/L.

Age (years)	Treatment	Clinical status	SAA (mg/L)	CRP (mg/L)	Proteinuria (g/24 h)	eGFR (mL/min)	Albumin (g/L)
13	Colchicine therapy initiated following diagnosis	Fever, abdominal pain, generalized edema; attacks every ~2 weeks	26	124	11	Preserved	22
~14	Escalated to anakinra 3 mg/kg/day after six months of persistent attacks on colchicine	Persistent recurrent fever and abdominal pain despite colchicine	--	--	--	--	--
15	Anakinra 3 mg/kg/day	Partial response; attack frequency reduced to ~2 episodes per year	18	4	--	--	--
16	Anakinra 3 mg/kg/day	Recurrent abdominal pain, peritoneal effusion; incomplete disease control	22	4	4	86	18
16+	Escalated to canakinumab 150 mg SC every four weeks	Follow-up ongoing	--	--	--	--	--

## Discussion

FMF is a monogenic autoinflammatory disease caused by mutations in the *MEFV* gene, characterized by recurrent episodes of fever and serositis, with a predilection for Mediterranean populations. Dysfunction of the pyrin protein encoded by MEFV leads to inappropriate activation of IL-1 and recurrent inflammatory episodes [1].

FMF is typically diagnosed during childhood; however, its clinical presentation may be misleading, particularly when abdominal attacks mimic acute surgical conditions. In untreated or late-diagnosed patients, amyloidosis may be the initial manifestation of the disease, often presenting as proteinuria or nephrotic syndrome [1].

AA amyloidosis represents the most severe complication of FMF and is a major cause of morbidity and mortality. Renal involvement is the most common presentation and may progress to end-stage renal disease if not adequately controlled [2]. The pathogenesis is directly linked to persistent elevation of SAA, an acute-phase reactant that serves as the precursor of amyloid fibrils. Sustained elevation of SAA is considered a prerequisite for the development of amyloidosis, highlighting the importance of tight inflammatory control [3].

Genetic factors play a crucial role in disease severity. The homozygous M694V mutation, identified in our patient, has consistently been associated with a more severe phenotype, earlier disease onset, increased frequency of attacks, and a higher risk of amyloidosis [4]. Interestingly, our case also illustrates intrafamilial phenotypic variability, as a sibling carrying the same homozygous mutation presented with a milder disease course controlled with colchicine alone, while another heterozygous sibling remained asymptomatic. This variability has been well described in the literature and is thought to result from the interaction between genetic, epigenetic, and environmental factors [5].

Colchicine remains the cornerstone of FMF treatment and is highly effective in preventing inflammatory attacks and amyloidosis when initiated early [1,6]. However, approximately 5-10% of patients exhibit colchicine-resistant disease, defined by persistent clinical attacks and/or elevated inflammatory markers despite adequate dosing and adherence [6]. Our patient met the criteria for colchicine resistance, with persistent symptoms after six months of therapy, necessitating escalation to biologic treatment.

IL-1 inhibitors have emerged as the main therapeutic option in colchicine-resistant FMF. Anakinra, a recombinant IL-1 receptor antagonist, has demonstrated efficacy in reducing attack frequency and inflammatory markers [6]. However, some patients may exhibit an incomplete response or develop persistent subclinical inflammation, as observed in our case.

A particularly important aspect of this case is the discordance between normal CRP levels and persistently elevated SAA. While CRP is commonly used in clinical practice, it may not reliably reflect ongoing inflammation in FMF. In contrast, SAA is a more sensitive biomarker and plays a direct role in amyloid deposition. Current recommendations emphasize the importance of monitoring SAA levels, with the goal of maintaining values below 10 mg/L to prevent amyloidosis progression [3]. Therefore, persistent elevation of SAA despite normal CRP should prompt therapeutic reassessment.

In this context, treatment was escalated to canakinumab, a long-acting monoclonal antibody targeting IL-1β. Canakinumab has demonstrated high efficacy in patients with colchicine-resistant FMF, including those previously treated with anakinra, leading to sustained remission and normalization of inflammatory markers in the majority of cases [7]. Furthermore, IL-1 inhibitors have been shown to reduce proteinuria and stabilize renal function in patients with FMF-associated amyloidosis [8].

Finally, this case underscores the importance of family screening in FMF, particularly in populations with high rates of consanguinity. Identification of asymptomatic or mildly affected individuals allows early initiation of colchicine therapy, which remains the most effective strategy for preventing amyloidosis and improving long-term outcomes.

## Conclusions

This case highlights the consequences of delayed diagnosis of FMF, which may lead to preventable complications such as AA amyloidosis. It underscores the importance of considering FMF as a potential diagnosis in patients with recurrent abdominal pain to avoid misdiagnosis and unnecessary surgery.

The presence of a homozygous M694V mutation and intrafamilial variability illustrates the heterogeneity of disease expression. Persistent elevation of SAA despite normal CRP in this case emphasizes its potential role in detecting ongoing subclinical inflammation.

This case suggests that early recognition, family screening, and timely therapeutic escalation, including IL-1 inhibitors, may contribute to improved outcomes and help prevent irreversible organ damage.
